# Bullying, cyberbullying, and social network addiction among adolescents with typical and non-typical development in Omani schools

**DOI:** 10.3389/fpsyg.2026.1865563

**Published:** 2026-09-17

**Authors:** Ahmed Al Saidi, Laura Badenes-Ribera, Antonio Ferrer, Watfa Al-Mamari, Paolo Bozzato, Sofia Mastrokoukou, Claudio Longobardi

**Affiliations:** 1 Department of Methodology of the Behavioral Sciences, Faculty of Psychology and Speech Therapy, University of Valencia, Valencia, Spain; 2 Child Health Department, College of Medicine and Health Sciences, Sultan Qaboos University, Muscat, Oman; 3 Department of Child Health, Sultan Qaboos University Hospital, University Medical City, Al-Khoudh, Oman; 4 Department of Developmental and Educational Psychology, Faculty of Psychology and Speech Therapy, University of Valencia, Valencia, Spain; 5 Department of Human Sciences and Innovation for the Territory, University of Insubria, Varese, Italy; 6 Department of Psychology, University of Turin, Turin, Italy

**Keywords:** adolescence, bullying, cyberbullying, non-typical development, Oman, social network addiction

## Abstract

**Introduction:**

Research on the prevalence of school bullying, cyberbullying, and social network addiction in Arab countries is limited. In Oman, these issues are of significant concern due to the country’s unique sociocultural context.

**Methods:**

This cross-sectional study examines the prevalence and associations between bullying, cyberbullying, and social network addiction among Omani adolescents with typical development (TD) and non-typical development (NTD). The study included a convenience sample of 1,458 students (50.2% female; mean age = 14.04 years; SD = 0.20) from grades 9-10 in public schools. Data were collected using the Adolescent Peer Relations Instrument, the Revised Cyber Bullying Inventory-II, and the Social Network Addiction Scale.

**Results:**

Findings revealed high levels of traditional bullying (35.9% perpetration; 53.5% victimization), with verbal aggression being the most prevalent form of both perpetration and victimization. Cyberbullying rates were moderate (9.7% perpetration; 19.9% victimization), consistent with international and regional data. Furthermore, 28.5% of adolescents met the study criterion for high social network addiction scores. Logistic regression analyses indicated that adolescents with NTD were more likely to report verbal bullying perpetration and verbal/social victimization, but less likely to report physical and social bullying perpetration, cybervictimization, or to meet the study criterion for high social network addiction scores than their TD peers. Significant cross-sectional indirect associations between traditional bullying and cyberbullying outcomes through problematic social network use were observed in the TD group, whereas the corresponding indirect associations were not statistically significant in the NTD group.

**Conclusion:**

These findings underscore the need for tailored prevention strategies that address different patterns of association in adolescents with diverse developmental profiles.

## Introduction

Adolescence is a critical developmental period characterized by rapid physical, cognitive, and social changes that influence identity formation and interpersonal relationships (Erikson, 1968; Schwartz and Pantin, 2006; Steinberg, 2020). While most adolescents progress along a typical developmental trajectory—herein referred to as typical development (TD), indicating normative progression in cognitive, social, and emotional milestones for their age group—a considerable proportion experiences non-typical development (NTD).

In the context of this study, the NTD group encompasses adolescents who have been formally identified as having neurodevelopmental disorders, specifically including autism spectrum disorder (ASD), attention-deficit/hyperactivity disorder (ADHD), learning disorders (LDs), childhood-onset fluency disorder, and other specified neurodevelopmental conditions. These conditions can often be associated with challenges in social communication, executive functions (including impulse regulation), language, behavioral regulation, and emotional understanding, potentially increasing their vulnerability to negative peer interactions and psychosocial maladjustment (American Psychiatric Association, 2022; Maïano et al., 2016).

This article presents an original study, conducted on a large sample of Omani adolescents, aiming to explore three interconnected phenomena: bullying, cyberbullying, and social network addiction in adolescents with TD and those with NTD.

### Bullying among adolescents

Bullying is a pervasive issue affecting adolescents worldwide, characterized by repeated aggressive behavior intended to harm or intimidate others (Olweus, 1993). This behavior can manifest in various forms, including physical aggression (e.g., direct bodily harm), verbal aggression (e.g., name-calling and insults), and relational aggression (e.g., social exclusion or actions aimed at damaging an individual’s social relationships or reputation) (Rivers and Smith, 1994). Victims may experience significant emotional and psychological distress, which can lead to decreased academic performance, low self-esteem, and even long-term mental health issues (Espelage and Swearer, 2003; Montes et al., 2022).

International estimates of bullying vary considerably because of differences in definitions, measurement instruments, and frequency thresholds (Biswas et al., 2020; Solberg and Olweus, 2003). In the 2021/2022 HBSC survey, approximately 6% of adolescents reported bullying others and 11% reported being bullied at least two or three times per month (Cosma et al., 2024). Available evidence suggests higher rates in Oman, with estimates of victimization generally ranging from approximately 38 to 54% across studies (Biswas et al., 2020; Fleming and Jacobsen, 2010; Peyton et al., 2017). Al-Saadoon et al. (2014) also documented a high prevalence of bullying involvement among Omani students, with verbal bullying emerging as the most common form. Although estimates are not directly comparable because of methodological differences, these findings indicate that bullying represents a substantial concern in Omani schools. Research suggests that bullying in Omani schools is influenced by several factors, including cultural values, peer relationships, and school environment (Kazarian and Ammar, 2013). Traditional values and norms, which emphasize respect for authority and seniority, can sometimes inadvertently support the power imbalances that underlie bullying behavior (Kooli, 2019). Moreover, reluctance to discuss personal issues openly due to cultural stigmas may prevent victims from seeking help, thereby perpetuating the cycle of abuse (Al Omari et al., 2022).

To our knowledge, no studies have specifically addressed the prevalence and characteristics of bullying in adolescents with NTD in Oman. This constitutes a significant gap, especially given international evidence indicating that adolescents with psychological disabilities or special education needs are disproportionately affected. A growing body of research consistently shows that these youths are more likely to be involved in bullying—either as victims, perpetrators, or both—compared to their typically developing peers (Abregú-Crespo et al., 2024; Berchiatti et al., 2022; Simmons and Antshel, 2021; Bitsika and Sharpley, 2014). For instance, a recent meta-analysis of 135 studies (Abregú-Crespo et al., 2024) reported that young people with mental health and/or neurodevelopmental conditions had 2.42 times greater odds of being perpetrators and 3.66 times greater odds of being both perpetrators and victims. These patterns may be due to their misunderstandings in social interactions, their low social competence, or as a reaction to being victimized themselves (Maïano et al., 2016).

### Cyberbullying among adolescents

With the advent of digital technology, a new form of bullying has emerged: cyberbullying. This phenomenon involves the use of electronic communication tools to harass, threaten, or humiliate others. Cyberbullying can occur through social media platforms, messaging apps, online games, and other digital mediums. Unlike traditional bullying, cyberbullying can be pervasive and persistent, with victims often feeling that there is no escape from their tormentors, as it often occurs beyond school grounds and outside of school hours (Barlett et al., 2023). Victims often experience increased anxiety, depression, and feelings of isolation, which can lead to low self-esteem, academic difficulties, antisocial action and even suicidal thoughts (Albikawi, 2023).

Recent HBSC data indicate prevalence rates of approximately 12% for cyberbullying perpetration and 16% for cybervictimization, with boys generally reporting higher perpetration rates, while gender differences in victimization vary across countries (Cosma et al., 2024). Estimates from Arab populations differ considerably, ranging from 14.8% among adolescents in Dubai to 42.8% in Saudi Arabia, partly reflecting methodological and contextual differences (Alhur and Alhur, 2023; Farooq et al., 2023). National evidence from Oman remains limited. Al-Shaqsi et al. (2022) reported 1,479 officially recorded cases in 2017, although administrative records are likely to underestimate unreported experiences. Therefore, standardized research on cyberbullying among Omani adolescents is still needed. International literature indicates that the likelihood of victimization is particularly high among adolescents with NTD, making them more vulnerable than their TD peers (Beckman et al., 2020). However, as claimed by Beckman et al. (2020), future research should verify this by including control groups of typically developing students and employing validated, standardized measures of cyberbullying. Additionally, there is limited evidence from Arab contexts regarding how cyberbullying victimization or perpetration patterns differ between TD and NTD adolescents.

### Social network addiction among adolescents

Social network sites have become an important context for adolescent communication, social interaction, and identity expression. While these platforms may offer opportunities for connectivity and access to information, excessive and poorly regulated use has been associated with problematic outcomes, including social network addiction (Andreassen, 2015; Andreassen and Pallesen, 2014). In the literature, the term social network addiction has often been used to describe excessive, poorly regulated, or compulsive patterns of social media use associated with negative consequences for mental health and daily functioning (Andreassen and Pallesen, 2014; Andreassen, 2015). However, this construct remains debated and should not be understood as a formal clinical diagnosis. Social network addiction is not currently recognized as a standalone disorder in major diagnostic classifications such as the DSM-5-TR or ICD-11; therefore, in the present study, the term refers to elevated self-reported symptoms of problematic social network use rather than to a clinically diagnosed addictive disorder. Drawing on social learning theory (Barlett, 2023) and the online disinhibition effect (Wu et al., 2023), excessive engagement with social media platforms has been theoretically linked to cyberbullying behaviors. From a social learning perspective, repeated exposure to aggressive online interactions may be associated with the normalization of hostility and the availability of behavioral models for imitation (Demircioğlu and Göncü-Köse, 2022). Concurrently, the asynchronous and often anonymous nature of digital communication may be associated with reduced interpersonal cues, lower perceived accountability, and greater online disinhibition (Hassan et al., 2023). These frameworks offer possible explanations for the association between problematic social media use and cyberbullying; however, they do not by themselves establish its temporal or causal direction.

A meta-analysis conducted across 32 countries found substantial cross-cultural variation in social network addiction, with prevalence estimates ranging from 14% in individualistic cultures to 31% in collectivistic cultures (Cheng et al., 2021). Evidence concerning Omani adolescents remains limited. Although Al-Badi et al. (2016), Shaikh (2024) documented extensive daily Internet use among Omani teenagers, time spent online does not necessarily indicate social network addiction. Research is therefore needed to estimate problematic use using validated measures and to examine its associations with adolescents’ offline and online peer experiences.

While previous research has primarily focused on dispositional factors (e.g., personality traits, low self-esteem, high need for belonging) as predictors of social network addiction, socio-cultural factors are also increasingly recognized (Andreassen and Pallesen, 2014). As in other Arab countries, social network addiction among adolescents in Oman may be associated with peer pressure, the need for social validation, and the use of online environments as an escape from academic and social stressors (Rabaa'i et al., 2018).

Regarding the influence of gender on social network addiction, existing research presents conflicting findings (Andreassen, 2015). Some studies suggest that males have higher addiction rates, while others emphasize that females are more prone to addiction. Other studies have found social network addiction unrelated to gender. These divergent findings may be influenced by a variety of factors, including cultural context, the types of social media platforms used, and the specific demographics of the sample studied. Importantly, in the context of Oman, where social norms and expectations often place different pressures on males and females, gender could influence the prevalence of social network addiction.

Empirical research indicates that social networking addicts tend to experience health-related, emotional, and relational problems (Andreassen, 2015). Social network addiction has been associated with sleep and somatic problems, depression and anxiety, lower self-esteem and life satisfaction, and poorer parent–child relationship quality (Andreassen, 2015; Cerutti et al., 2016; Hawi and Samaha, 2017; Li et al., 2018; Shafiq et al., 2023; White-Gosselin and Poulin, 2024).

In Oman, research on social network addiction has mainly focused on university students (Bani-Hamad and Jaradat, 2022), whereas adolescents have received limited research attention. Evidence on teenagers’ Internet behavior suggests extensive daily Internet use among Omani adolescents (Al-Badi et al., 2016; Shaikh, 2024), although time spent online should not be equated with social network addiction.

Additionally, limited research has focused on social network addiction among adolescents with NTD. Even though studies vary in their results, it appears that the time spent on social media is comparable between adolescents with TD and those with NTD (Alhujaili et al., 2022). However, adolescents with ASD differ from their typical peers in both their preferred social media sites and their reasons for use. While individuals with TD use social media primarily as a means of interaction, those with ASD are more likely to use social media for entertainment purposes (Alhujaili et al., 2022).

### Bullying, cyberbullying, and social network addiction

A growing body of evidence suggests that adolescents involved in traditional bullying and cyberbullying (whether as victims or perpetrators) show elevated levels of problematic social network site use (Martínez-Ferrer et al., 2018; Longobardi et al., 2020). Victimized adolescents may turn to social networks as a coping strategy to escape negative affect and stress, finding in these platforms virtual spaces that offer emotional relief, a sense of belonging, and opportunities to restore damaged self-esteem (Boniel-Nissim and Sasson, 2018; Etgar and Amichai-Hamburger, 2017). However, this reliance may be associated with compulsive use, emotional dysregulation, withdrawal from other life activities, and reduced behavioral control (Zimmer-Gembeck et al., 2021).

Additionally, adolescents who spend more time on social networks are more likely to engage in deviant online behavior, including cyberbullying (Giordano et al., 2021; Kırcaburun et al., 2019). This link has been explained by frameworks such as the problem behavior theory (Jessor, 1991), which posits that adolescents engaging in one risk behavior are more prone to engage in others, and the general strain theory of Internet addiction, which proposes that emotional stress associated with compulsive social media use may also be related to aggression in online contexts (Mubarak and Quinn, 2019).

In this context, social network addiction has been proposed as a potential mediator between traditional bullying experiences (as perpetrator or victim) and cyberbullying. Adolescents who experience or perpetrate traditional bullying may also report problematic social media use, and both experiences may be associated with cyberbullying involvement. Problematic social media use has therefore been examined as a potential statistical intermediary in the association between traditional bullying and cyberbullying outcomes. Martínez-Ferrer et al. (2021), for example, reported significant indirect associations between traditional victimization and both cybervictimization and cyberbullying through problematic social network site use. Such findings are consistent with a possible explanatory process linking offline and online experiences; however, the temporal and causal ordering of these variables requires longitudinal confirmation. These indirect associations remain underexplored in non-Western contexts, particularly in Oman, where social and technological dynamics differ substantially from those in Western settings.

### The current study

The present study aims to fill this gap by examining the prevalence, group differences, and associations between bullying, cyberbullying, and social network addiction among a large sample of Omani adolescents with either typical development (TD) or non-typical development (NTD). This is one of the first studies in the region to adopt this comparative approach. Specifically, it addresses three research questions, each accompanied by testable hypotheses:

*Research Question 1*: What is the prevalence of bullying, cyberbullying, and social network addiction among Omani adolescents?

*Hypothesis 1*: Drawing from the literature review, we hypothesized that prevalence rates of bullying, cyberbullying, and social network addiction scores would be elevated. We also expected verbal aggression to be the most prevalent form of both bullying perpetration and victimization.

*Research Question 2*: Do adolescents with TD and NTD differ in bullying and cyberbullying perpetration and victimization, as well as in levels of social network addiction, after controlling for sociodemographic characteristics (gender, age, family income, and parental education)?

*Hypothesis 2*: Adolescents with NTD will report significantly higher levels of victimization in both bullying and cyberbullying compared to their TD peers.

*Hypothesis 3*: Adolescents with TD will report higher rates of bullying and cyberbullying perpetration than adolescents with NTD.

*Hypothesis 4*: Adolescents with NTD will exhibit significantly higher social network addiction scores than adolescents with TD.

*Hypothesis 5*: Gender differences will emerge such that boys report higher rates of physical bullying perpetration, whereas girls report higher rates of relational bullying perpetration and cyberbullying victimization.

*Research Question 3*: Are traditional bullying perpetration and victimization indirectly associated with cyberbullying perpetration and victimization through social network addiction among adolescents with typical and non-typical development?

*Hypothesis 6*: Bullying perpetration and victimization will be positively associated with social network addiction and with both cyberbullying perpetration and victimization.

*Hypothesis 7*: Social network addiction will be positively associated with both cyberbullying perpetration and victimization.

*Hypothesis 8*: Bullying perpetration and victimization will show statistically significant indirect associations with cyberbullying perpetration and victimization through social network addiction. These hypothesized indirect associations refer to the specified statistical model and do not imply temporal or causal ordering.

This study contributes to the limited body of empirical work on adolescent psychosocial risk in the Arab region and provides culturally grounded insights into the intersection of developmental status, peer aggression, and digital behavior. The findings may inform prevention and intervention strategies targeting vulnerable youth in both educational and clinical settings.

## Method

### Participants

A cross-sectional study was conducted using a convenience sample composed of 1,458 students (732 females) recruited from public schools in grade 9 and grade 10 in the Governorate of Muscat, the capital of Oman. Participants’ mean age was 14.04 years (*SD* = 0.20, range = 14–15). Of them, 1,339 were adolescents with TD (50.5% females) and 119 were adolescents with NTD (47.1% females). Within the NTD group, 51 adolescents had autism spectrum disorder (42.9%), 28 had attention-deficit/hyperactivity disorder (23.5%), 21 had a specific learning disability (17.6%), 14 had childhood-onset fluency disorder (stuttering; 11.8%), and five had another specified neurodevelopmental condition (4.2%). The mean age for adolescents with TD was 14.05 (*SD* = 0.21) and for adolescents with NTD was 14.03 (*SD* = 0.16). There were no statistically significant differences between adolescents with TD and adolescents with NTD in age [*Welch’s t*(157.36) = 1.311, *p* = 0.192, *Cohen’s d* = 0.10, 95% CI (−0.09, 0.29)] or in gender distribution (*χ^2^* [1] = 0.51, *Phi coefficient* = −0.02; *p* = 0.474).

A total of six public schools participated in the study. Schools were selected in collaboration with the Omani Ministry of Education, based on their geographic accessibility, enrollment size, and administrative willingness to support the research. All selected schools were located in urban districts of Muscat, ensuring consistency in terms of infrastructural resources and digital access. The participating schools were selected through convenience sampling to ensure the feasibility of data collection rather than to obtain a nationally representative sample. Consequently, adolescents attending private schools and schools located in rural governorates were not represented in the present study. Table 1 displays sociodemographic characteristics of the participants’ parents. Overall, most parents lived together (99.7%) and had completed secondary education. The most frequent monthly income was between 901–1,200 Omani Rials.

**Table 1:** Sociodemographic characteristics of participants’ parents.

Characteristic	Total (*N* = 1,458)	Typical development (*n* = 1,339)	Non-typical development (*n* = 119)
	*n* (%)	*n* (%)	*n* (%)
Father education			
Cannot read or write	26 (1.8)	26 (1.9)	–
Primary	156 (10.7)	145 (10.8)	11 (9.2)
Secondary	1,087 (74.6)	1,004 (75.0)	83 (69.7)
College	135 (9.3)	110 (8.2)	25 (21.0)
Postgraduate	54 (3.7)	54 (4.0)	–
Mother education			
Cannot read nor write	8 (0.5)	8 (0.6)	–
Primary	41 (2.8)	40 (3.0)	1 (0.8)
Secondary	1,250 (85.7)	1,152 (86.0)	98 (82.4)
College	157 (10.8)	137 (10.2)	20 (16.8)
Postgraduate	2 (0.1)	2 (0.1)	–
Monthly income (Omani Rials)			
325–500	45 (3.1)	44 (3.3)	1 (0.8)
501–750	354 (24.3)	333 (24.9)	21 (17.6)
751–900	–	–	–
901–1,200	734 (50.3)	676 (50.5)	58 (48.7)
1,201 and above	325 (22.3)	286 (21.4)	39 (32.8)
Parents marital status			
Live together	1,453 (99.7)	1,334 (99.6)	119 (100)
Divorced	5 (0.3)	5 (0.4)	–

### Measures

#### Socio-demographic characteristics

Participants were asked to report on the socio-demographic information: current age, gender, father’s and mother’s educational levels, monthly household income (Omani Rials), and parents’ marital status.

#### Translation and cultural adaptation of the instruments

All questionnaires were administered in Arabic. The Addiction to Social Network Scale was originally developed in Arabic (Younis, 2016), whereas a previously validated Arabic version of the Revised Cyber Bullying Inventory-II (RCBI-II) was used (Sahli et al., 2023). Because no validated Arabic version of the Adolescent Peer Relations Instrument (APRI) was available, the instrument was translated into Arabic following internationally accepted guidelines for the cross-cultural adaptation of self-report measures (Beaton et al., 2000; Wild et al., 2005). Two independent bilingual translators translated the original English version into Arabic. The two translations were subsequently compared and reconciled into a single preliminary version through discussion and consensus. This version was then back-translated into English by an independent bilingual translator who was blinded to the original instrument. The original, translated, and back-translated versions were reviewed by the research team to identify and resolve discrepancies, ensuring conceptual rather than literal equivalence. The preliminary Arabic version was subsequently evaluated by an expert panel comprising specialists in developmental psychology, educational psychology, special education, and psychometrics with experience in the Omani educational context. The panel assessed the linguistic clarity, cultural appropriateness, and conceptual equivalence of each item. Minor linguistic refinements were introduced to improve comprehensibility while preserving the conceptual content, response format, and scoring procedures of the original instrument. The translated version was then pilot tested with a small group of Omani adolescents representative of the target population to evaluate item clarity, comprehensibility, and completion time. Feedback obtained during pilot testing resulted only in minor wording refinements, and no modifications were made to the conceptual content or scoring of the instrument. The reliability coefficients reported for the present sample further support the internal consistency of the Arabic instruments administered in this study.

#### Presence of non-typical development in adolescents (NTD)

The identification of adolescents with non-typical development (NTD) was based on official school records and teacher reports, following the guidelines set by the Omani Ministry of Education for classifying students with disabilities or special educational needs (SEN). In Oman, the eligibility for SEN classification adheres to diagnostic criteria based on the DSM-5-TR, and requires formal assessment by licensed health professionals, including psychologists, psychiatrists, or pediatricians. Diagnoses that qualify students for SEN services—and thus inclusion in the NTD category-include: learning disabilities (LD), autism spectrum disorder (ASD), attention-deficit/hyperactivity disorder (ADHD), intellectual disability (ID), speech and language disorders, and other neurodevelopmental delays.

All schools in Oman are required to maintain a SEN Student Profile for identified students. This profile includes copies of medical diagnostic reports, psychological assessments, and individualized education plans (IEPs). Teachers participating in this study received a standardized protocol guiding them to verify whether a student: (1) was formally identified in the school’s SEN registry, and (2) had a confirmed diagnosis recorded in the school’s documentation, supported by a licensed health provider.

Teachers selected for this study had undergone annual training organized by the Ministry’s Special Education Department, covering disability identification, classroom accommodations, and referral procedures. These teachers were also familiar with the Resource Room Programs (RRP) used across Muscat schools, which provide targeted academic and behavioral interventions for students with SEN. Data about each participant’s SEN status was cross-validated with the school’s SEN coordinator before final classification.

For the purpose of analysis, a binary variable was created: adolescents with typical development (without a documented neurodevelopmental condition) were coded as “0” (Typical Development, TD), and those with a verified SEN diagnosis as “1” (Non-Typical Development, NTD). This operational definition allowed for clear differentiation based on documented diagnostic and institutional criteria. The database recorded one primary diagnostic category for each participant. Therefore, information regarding co-occurring diagnoses and time since diagnosis were not available. The diagnostic composition of the NTD group is reported in the Participants section.

Although the NTD group comprised adolescents with different neurodevelopmental conditions, the decision to operationalize these participants as a single analytical group was consistent with the primary objective of the present study, namely, to compare adolescents with and without formally documented neurodevelopmental conditions within mainstream educational settings. Because the diagnosis-specific subgroups were small and unequal in size, the analyses were conducted using the binary TD–NTD classification. Accordingly, the findings should be interpreted as representing aggregate patterns across a diagnostically heterogeneous NTD group and should not be assumed to apply uniformly to each individual diagnostic category.

National estimates indicate that approximately 7–9% of adolescents in Oman qualify as having SEN under Ministry guidelines, with the majority diagnosed with LD, followed by ASD and ADHD. The proportion of adolescents with NTD in the present study (119 out of 1,458; ~8.2%) is consistent with these national prevalence rates.

#### Adolescent peer relations instrument

The APRI is a widely used self-report instrument designed to assess bullying perpetration and victimization among adolescents (APRI; Parada, 2000). It has demonstrated good psychometric properties (reliability and validity) across different cultural contexts (e.g., Balan et al., 2022; Choudhary et al., 2024; Marsh et al., 2011). Because no validated Arabic version of the APRI was available, the instrument was translated and culturally adapted into Arabic following the procedure described above. The APRI consists of 36 items which measure the frequency during the last school year of three types of behaviors used to bully others (physical, verbal, and social) and three ways of being targeted (physical, verbal, and social). Each item is rated on a six-point Likert response scale (1 = never, 2 = sometimes, 3 = once or twice a month, 4 = once a week, 5 = several times a week, 6 = every day). Responses closer to 1 represented small amounts of bullying or being bullied, whereas scores closer to 6 represented frequent amounts of bullying or being bullied. The scores for each of the six subscales were generated by summing the scores for the items that made up that scale. Each six-item form-specific subscale ranges from 6 to 36. The total perpetration and total victimization scores, each comprising 18 items, range from 18 to 108. Higher scores indicate higher levels of bullying behavior or victimization. For the present study, internal consistency reliability was evaluated using both McDonald’s omega (*ω*) and Cronbach’s alpha (*α*). Reliability was satisfactory for the total bullying perpetration scale (*ω* = 0.887, *α* = 0.885) and the total victimization scale (*ω* = 0.924, *α* = 0.922), as well as for all APRI subscales: Physical bullying (*ω* = 0.737, *α* = 0.721), Verbal bullying (*ω* = 0.790, *α* = 0.777), Social bullying (*ω* = 0.747, *α* = 0.742), Physical victimization (*ω* = 0.792, *α* = 0.793), Verbal victimization (*ω* = 0.839, *α* = 0.813), and Social victimization (*ω* = 0.834, *α* = 0.834).

To classify participants for prevalence analyses, the operational definition of bullying proposed by Olweus (1993) and Solberg and Olweus (2003) was adopted. This definition, which identifies bullying involvement as behaviors occurring at least once or twice a month, has been widely used in international epidemiological research and has also been applied in studies conducted among Arab adolescents (e.g., Al-Saadoon et al., 2014; Biswas et al., 2020).

To meet the repetition criterion central to the definition of bullying (e.g., Olweus, 1993; Solberg and Olweus, 2003), participants were categorized as being “involved” in bullying perpetration or victimization if they reported experiencing or engaging in at least one form of bullying behavior at a frequency of once or twice a month (≥ 3 on any item of the respective subscale). Conversely, participants who reported no involvement or only occasional involvement (≤ 2 on all items) were classified as “not involved.”

#### Revised cyber bullying inventory-II

The RCBI-II is a psychometric tool designed to assess both cyberbullying and cybervictimization behaviors among adolescents (RCBI-II; Topcu and Erdur-Baker, 2018). A previously validated Arabic version of the instrument was administered (Sahli et al., 2023). The instrument consists of 10 items, each rated twice by participants: once in the “I did it” column to report perpetration of cyberbullying and once in the “It happened to me” column to report experiences of cybervictimization. Responses are provided on a 4-point scale, with the following options: 1 = never, 2 = once, 3 = twice or three times, 4 = more than three times. Total scores for each dimension range from 10 to 40, with higher scores indicating greater frequency of cyberbullying perpetration or cybervictimization.

For the present study, internal consistency reliability was evaluated using both McDonald’s omega (ω) and Cronbach’s alpha (α). Reliability was satisfactory for both the Cyberbullying scale (ω = 0.852, α = 0.858) and the Cybervictimization scale (ω = 0.853, α = 0.851).

The original scoring procedure and classification criteria proposed by the developers of the instrument (Topcu and Erdur-Baker, 2018) were retained in the present study. The same scoring procedure was also adopted in the validated Arabic version of the RCBI-II (Sahli et al., 2023), thereby ensuring consistency with previous applications of the instrument in Arabic-speaking adolescent populations.

For analytical purposes, participants were subsequently classified into dichotomous categories (e.g., Olweus, 1993; Solberg and Olweus, 2003). Participants were categorized as being “involved” in cyberbullying perpetration or cybervictimization if they reported engaging in or experiencing at least one form of cyberbullying behavior at a frequency of twice or three times (≥ 3 on any item of the respective subscale). Conversely, participants who reported no involvement or only occasional involvement (≤ 2 on all items) in cyberbullying perpetration or cybervictimization were classified as “not involved.”

Participants classified as being involved in both cyberbullying perpetration and cybervictimization were subsequently categorized as cyberbully-victims using the dichotomized scores of the two subscales.

#### The addiction to social network scale

Younis (2016) was employed to measure the intensity of adolescents’ engagement with social networking platforms. Because the instrument was originally developed in Arabic, no linguistic or cultural adaptation was required for the present study. It consists of 30 items rated on a 3-point Likert scale (0 = no, 1 = sometimes, 2 = yes). A total score was computed by summing all items. Higher scores indicate greater levels of problematic social network use.

For this study, internal consistency reliability was evaluated using both McDonald’s omega (*ω*) and Cronbach’s alpha (*α*). Reliability was excellent (*ω* = 0.911, *α* = 0.912). For prevalence and logistic regression analyses, participants’ scores were dichotomized using the 75th percentile as the cut-off. In the absence of universally accepted clinical or diagnostic thresholds for problematic social network use among adolescents, this sample-based threshold was selected to identify participants reporting relatively elevated levels of problematic social network use.

Participants scoring at or above the 75th percentile were classified as belonging to the high social network addiction group (1), whereas those scoring below this threshold were classified as belonging to the lower-score group (0). This categorization was adopted exclusively for analytical purposes and should not be interpreted as indicating a clinical diagnosis of addiction. Rather, it identifies adolescents reporting relatively elevated levels of problematic social network use within the present sample. Although dichotomization inevitably reduces statistical variability (MacCallum et al., 2002), this approach facilitated prevalence estimation and was consistent with the objectives of the present study. This approach aligns with previous research using percentile-based cut-offs to identify individuals with relatively elevated levels of problematic digital-media use (e.g., Kuss et al., 2014).

### Procedure and ethics considerations

Prior to participation in the study, informed consent was obtained from both the students and their parents or legal guardians. The participants were students enrolled in grades 9 and 10. The data collection process was conducted within the classroom setting to ensure convenience and minimize disruption to the school routine. A trained research assistant was present to oversee the administration, provide clarification if needed, and ensure standardized implementation of the procedure across classrooms.

The data were collected using anonymous self-report questionnaires, which were distributed in printed form during a dedicated session agreed upon with school administrators. Students were informed that completion of the questionnaire was voluntary, anonymous, and would not affect their academic standing in any way. The estimated time required for each participant to complete the questionnaire was approximately 30 min, during which they were seated individually to reduce the possibility of peer influence or response bias.

The study protocol received ethical approval from the Institutional Review Board (IRB) of the University (Approval No. 0592463). The research procedures strictly complied with the ethical principles outlined in the Declaration of Helsinki. Particular attention was paid to ensure that no psychological or physical harm would result from participation in the study. Questions were carefully screened to avoid sensitive or distressing content, and students were given the option to skip any item they did not feel comfortable answering.

Confidentiality was safeguarded at all stages of the study. No identifying information was collected, and responses were stored securely with access limited to the research team. The data were anonymized and analyzed in aggregate form only. To further ensure the anonymity of the participants, each questionnaire was assigned a unique numerical identifier and no names or personal details appeared on any form. After completion, students placed their questionnaires in sealed envelopes, which were collected by the research assistant and stored in a locked cabinet accessible only to the principal investigator. The data remained anonymized throughout the analysis and was only reported in aggregated form. Participants were fully informed about the purpose of the study, the voluntary nature of their participation, their right to withdraw at any time without consequence, and the measures taken to protect their privacy. These procedures ensured adherence to ethical standards governing research with human participants, and contributed to a respectful and ethically sound research environment.

### Power analysis

An *a priori* power analysis was conducted in G*Power 3.1 (Faul et al., 2007) to establish the minimum sample size necessary for our primary inferential tests. For chi-square tests of independence (df = 1), a small effect size (*w* = 0.10; Cohen, 1988), *α* = 0.05, and statistical power (1 – *β*) of 0.80 yielded a required sample size of approximately 785 participants. For logistic regression analyses aimed at detecting an odds ratio of 1.50 assuming a predictor prevalence of 0.30 (two-tailed *α* = 0.05, 1 – *β* = 0.80), the estimated required sample size was approximately 820 participants. For two-tailed bivariate correlations, detecting a small association of *r* = 0.10 with *α* = 0.05 and power of 0.80 required approximately 783 participants. The final sample of *N* = 1,458 participants therefore exceeds these thresholds, ensuring ≥ 0.80 power to detect small-to-medium effects across all planned analyses.

### Nesting and cluster effects

Participants (*N* = 1,458) were nested within 56 classrooms across six urban public schools in Muscat, with an average classroom size of approximately 26 students. To quantify the degree of non-independence, intraclass correlation coefficients (*ICCs*) were estimated for each primary outcome (bullying victimization, bullying perpetration, cyberbullying victimization, cyberbullying perpetration, and social network addiction) using one-way random-effects ANOVA procedures (Raudenbush and Bryk, 2002). *ICC* values ranged from 0.01 to 0.04, indicating that between **1** and 4% of the total variance in the outcomes was attributable to differences between classrooms. Design effects (*DEFFs*) were calculated using the formula *DEFF* = 1 + (*m* − 1) × *ICC* (Kish, 1965), where *m* represents the average classroom size. Based on an average classroom size of approximately 26 students, the corresponding *DEFFs* ranged from 1.25 to 2.00, indicating low to modest clustering effects. Because the *ICC*s were small and the primary objective was to estimate population-average associations, single-level chi-square tests and logistic regression models were retained as the primary analyses. To evaluate the robustness of this approach, two-level generalized linear mixed models with random intercepts for classroom were also estimated. The multilevel analyses yielded parameter estimates that were highly comparable to those obtained from the single-level models, and the overall pattern of statistically significant findings remained unchanged, indicating that the substantive conclusions were robust to classroom-level clustering.

### Strategy of analysis

Data were double-entered and verified to ensure accuracy. The proportion of missing values was below 1% for all variables, with a maximum of 0.2% per variable. As such a low level of missingness is considered negligible and unlikely to bias parameter estimates, no imputation or adjustment procedures were applied to the observed data (Graham, 2009). In addition, internal consistency of the study measures was evaluated using both Cronbach’s alpha and McDonald’s omega coefficients, with values above 0.70 indicating acceptable reliability (Kline, 2016).

The analytical strategy proceeded as follows. First, descriptive statistics (means and standard deviations for continuous variables; frequencies and percentages for categorical variables) were computed to summarize participants’ characteristics. A chi-square test was conducted to assess differences in gender distribution between adolescents with typical development (TD) and those with non-typical development (NTD). Levene’s test was used to assess the assumption of homogeneity of variances for age. Because the test indicated unequal variances, *F*(1, 1,456) = 4.48, *p* = 0.034, Welch’s *t* test was used to compare mean age between the TD and NTD groups.

Second, because one of the primary objectives of the study was to estimate prevalence rates and compare the likelihood of involvement in bullying, cyberbullying, and elevated social network addiction between adolescents with TD and NTD, bullying, cyberbullying, and social network addiction variables were operationalized using established categorical thresholds. For bullying and cyberbullying, cut-offs consistent with the behavioral criterion of repeated aggression commonly adopted in epidemiological research were applied (Olweus, 1993; Solberg and Olweus, 2003). For social network addiction, a sample-based 75th-percentile threshold was used to identify adolescents reporting relatively elevated levels of problematic social network use, as no universally accepted diagnostic cut-off currently exists. Although dichotomization inevitably reduces variability and statistical information and may attenuate associations observed in continuous data (MacCallum et al., 2002), this approach was selected because the primary research questions focused on estimating prevalence and comparing the likelihood of involvement in bullying, cyberbullying, and elevated social network addiction between adolescents with TD and NTD. Moreover, the use of categorical classifications is consistent with previous epidemiological studies examining bullying involvement using comparable behavioral thresholds (Olweus, 1993; Solberg and Olweus, 2003). Differences in the prevalence of bullying behaviors across forms (verbal, social, and physical) were assessed using Cochran’s *Q* test for related samples.

Third, binary logistic regression analyses were conducted to examine group differences in bullying perpetration, victimization, cyberbullying, and social network addiction between adolescents with typical development (TD) and those with non-typical development (NTD). All models were adjusted for relevant covariates, including participant age and gender, as well as parental educational level and household income. Odds ratios (OR) with 95% confidence intervals (CI) were reported as measures of effect size.

Fourth, bivariate correlations were computed to explore the relationships among the study variables. Subsequently, a path model was specified to examine the hypothesized direct and indirect associations between traditional bullying (perpetration and victimization), social network addiction, and cyberbullying (perpetration and victimization) (see Figure 1). In the specified model, traditional bullying perpetration and victimization were included as predictors, social network addiction as the statistical intermediary, and cyberbullying perpetration and cybervictimization as outcomes. Because all variables were assessed concurrently, the ordering of the variables in the model reflects the hypothesized conceptual structure and does not establish temporal precedence or causal mediation. The model was estimated using robust maximum likelihood estimation (MLR), with full information maximum likelihood (FIML) employed to address missing data. Moreover, the grouping function was used to estimate the model separately for adolescents with TD and adolescents with NTD. This approach permitted an examination of whether the pattern of associations among traditional bullying, social network addiction, and cyberbullying differed between the two groups. Covariates that were significantly associated with the outcome variables in prior binary logistic regression analyses (namely, age, father’s education, mother’s education, and family income) were included in the model to statistically control for their potential confounding influence.

**Figure 1:**
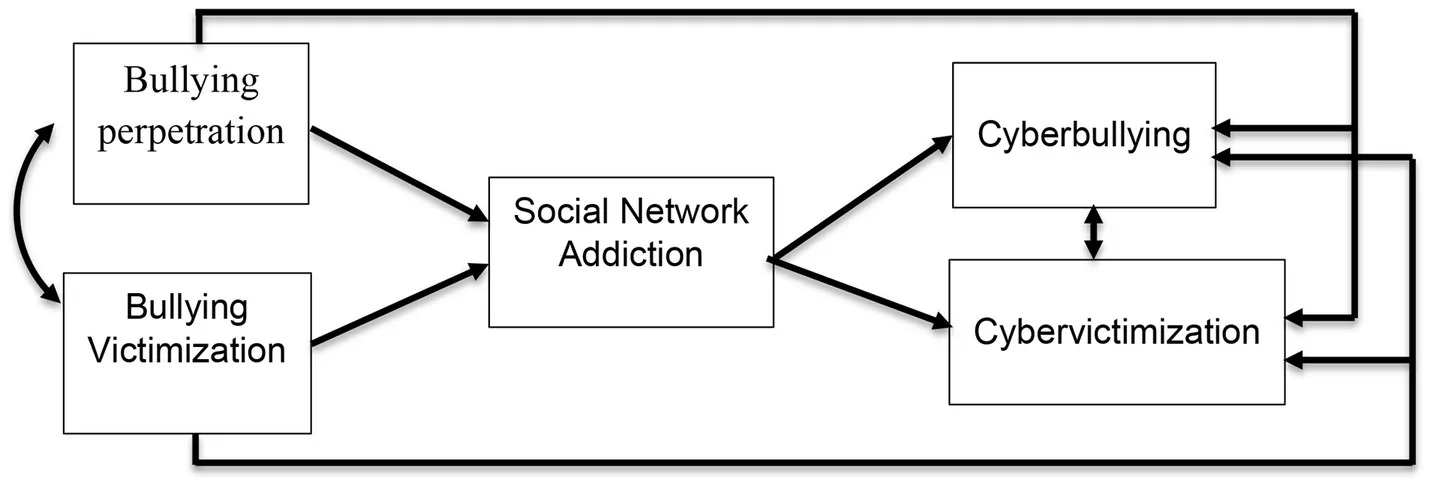
Theoretical model illustrating the hypothesized direct and indirect associations between traditional bullying (perpetration and victimization) and cyberbullying (perpetration and victimization), through problematic social network use.

Model fit was evaluated using several commonly recommended fit indices (Kline, 2016), including the Chi-square statistic (χ^2^), the Comparative Fit Index (CFI), the Root Mean Square Error of Approximation (RMSEA), and the Standardized Root Mean Square Residual (SRMR). Based on established guidelines (Hu and Bentler, 1999; Hooper et al., 2008), CFI values ≥ 0.90, RMSEA values ≤ 0.08, and SRMR values ≤ 0.08 were interpreted as indicative of acceptable model fit. Indirect effects were tested using bias-corrected bootstrapped 95% confidence intervals. An indirect effect was considered statistically significant if the 95% confidence interval did not include zero.

Descriptive statistics, logistic regression analyses and correlations among the variables were estimated in SPSS 28 (IBM Corporation, 2021), and the path model was estimated in Mplus 8.9 (Muthén and Muthén, 1998-2017). All analyses were interpreted at a significance level of 0.05.

## Results

### Prevalence of bullying, cyberbullying, and social network addiction

Table 2 presents the prevalence rates of bullying, cyberbullying, and social network addiction for the entire sample as well as for subgroups. As shown in the table, 35.9% of adolescents reported engaging in at least one form of bullying. Specifically, 25.3% of adolescents engaged in verbal bullying, 14.5% in social bullying, and 24.5% in physical bullying. Additionally, a higher proportion of adolescents (53.5%) reported being victims of bullying, with 41.6% experiencing verbal victimization, 36.1% social victimization, and 28.3% physical victimization.

**Table 2:** Prevalence of bullying (≥3 on any item of the respective subscale), cyberbullying (≥3 on any item of the respective subscale), and social network addiction (≥75th percentile) for total sample and for groups of adolescents with TD and NTD.

Outcome	Total (*N* = 1,458)	Typical development (*n* = 1,339)	Non-typical development (*n* = 119)
	*n* (%)	*n* (%)	*n* (%)
Bullying perpetration (APRI)			
Verbal	369 (25.3)	322 (24.0)	47 (39.5)
Social	211 (14.5)	201 (15.0)	10 (8.4)
Physical	357 (24.5)	342 (25.5)	15 (12.6)
Total (any form)	524 (35.9)	476 (35.5)	48 (40.3)
Bullying victimization (APRI)			
Verbal	607 (41.6)	537 (40.1)	70 (58.8)
Social	527 (36.1)	459 (34.3)	68 (57.1)
Physical	412 (28.3)	379 (28.3)	33 (27.7)
Total (any form)	780 (53.5)	696 (52.0)	84 (70.6)
Cyberbullying (RCBI-II)	142 (9.7)	133 (9.9)	9 (7.6)
Cybervictimization (RCBI-II)	290 (19.9)	287 (21.4)	3 (2.5)
Social network addiction	416 (28.5)	393 (29.4)	23 (19.3)

Adolescents with typical development = 0. Adolescent with non-typical development = 1. APRI, Adolescent Peer Relations Instrument; RCBI-II, Revised Cyber Bullying Inventory-II. Adolescents with typical development were coded 0; adolescents with non-typical development were coded 1.

In terms of cyberbullying, only 9.7% of participants admitted to perpetrating cyberbullying, while 19.9% reported being victims of cyberbullying. Furthermore, nearly one-third of the participants (28.5%) exhibited high scores on the social network addiction scale.

Cochran’s *Q* test was performed to assess differences in the prevalence of the various forms of bullying perpetration and victimization. The test revealed statistically significant differences in both bullying perpetration (*Q*(2) = 98.929, *p* < 0.001) and bullying victimization (*Q*(2) = 79.677, *p* < 0.001). *Post hoc* comparisons, adjusted for Bonferroni, indicated that verbal and physical bullying were the most prevalent forms of perpetration (both *p* < 0.001). No statistically significant difference was found between the prevalence of verbal and physical bullying perpetration (*p* = 0.577).

Regarding bullying victimization, verbal victimization was the most prevalent form (*p* < 0.001), while physical victimization was the least prevalent.

### Differences in bullying, cyberbullying, and social network addiction between adolescents with typical and non-typical development

Logistic regression analyses were conducted to examine group differences between adolescents with typical development (TD, coded as 0) and those with non-typical development (NTD, coded as 1) in terms of bullying perpetration, bullying victimization, cyberbullying, and social network addiction. The models were adjusted for participant age, gender, parental educational level, and household income. The results are displayed in Table 3.

**Table 3:** Results of multiple logistic regression analyses examining the association between developmental status and bullying, cyberbullying, and social network addiction outcomes, controlling for participants’ gender and age and parental characteristics.

Predictor	Perpetration				Victimization						
	Verbal	Social	Physical	Total	Verbal	Social	Physical	Total	Cyberbullying	Cyberbullied	SNA
	OR (95% CI)	OR (95% CI)	OR (95% CI)	OR (95% CI)	OR (95% CI)	OR (95% CI)	OR (95% CI)	OR (95% CI)	OR (95% CI)	OR (95% CI)	OR (95% CI)
Presence of disability (NTD)	2.114 (1.397, 3.198)***	0.495 (0.250, 0.980)*	0.350 (0.196, 0.624)***	1.213 (0.809, 1.819)	2.383 (1.584, 3.584)***	2.802 (1.891, 4.152)***	1.021 (0.665, 1.567)	2.341 (1.529, 3.584)***	0.632 (0.300, 1.331)	0.090 (0.028, 0.287)***	0.548 (0.335, 0.897)*
Sex (female)	1.054 (0.820, 1.354)	0.989 (0.708, 1.298)	0.936 (0.727, 1.206)	1.038 (0.829, 1.301)	1.029 (0.824, 1.287)	1.009 (0.810, 1.257)	1.066 (0.843, 1.347)	1.012 (0.815, 1.256)	1.223 (0.844, 1.772)	1.064 (0.815, 1.390)	0.948 (0.748, 1.203)
Age (15 years)	1.267 (0.654, 2.452)	2.082 (1.085, 3.994)*	1.143 (0.582, 2.244)	1.714 (0.988, 2.972)	0.755 (0.415, 1.372)	1 (0.574, 1.741)	0.393 (0.181, 0.854)*	0.668 (0.386, 1.157)	0.721 (0.211, 2.469)	0.101 (0.023, 0.436)**	1.322 (0.732, 2.389)
Father education											
Secondary	0.493 (0.296, 0.823)***	1.285 (0.642, 2.570)	0.604 (0.352, 1.036)	1.062 (0.659, 1.711)	0.834 (0.543, 1.283)	1.245 (0.811, 1.912)	0.782 (0.494, 1.238)	1.013 (0.674, 1.523)	1.049 (0.378, 2.913)	4.608 (2.141, 9.921)***	0.392 (0.256, 0.601)***
College & postgraduate	0.126 (0.063, 0.251)***	0.910 (0.393, 2.107)	0.160 (0.079, 0.324)***	0.312 (0.167, 0.583)***	0.136 (0.073, 0.254)***	0.362 (0.201, 0.655)***	0.330 (0.177, 0.616)***	0.299 (0.173, 0.514)***	0.377 (0.117, 1.221)	1.737 (0.696, 4.334)	0.087 (0.046, 0.167)***
Mother education											
Secondary	0.434 (0.209, 0.901)*	0.103 (0.045, 0.235)***	1.645 (0.638, 4.239)	0.176 (0.086, 0.360)***	0.129 (0.060, 0.278)***	0.411 (0.211, 0.802)**	0.188 (0.093, 0.381)***	0.188 (0.088, 0.401)***	0.096 (0.031, 0.298)***	0.101 (0.040, 0.253)***	0.740 (0.383, 1.433)
College & postgraduate	2.377 (1.023, 5.522)*	0.386 (0.152, 0.981)*	7.449 (2.651, 20.934)***	0.715 (0.314, 1.628)	0.678 (0.278, 1.652)	0.776 (0.358, 1.681)	0.195 (0.086, 0.442)***	0.466 (0.197, 1.106)	0.77 (0.227, 2.688)	0.275 (0.100, 0.758)*	4.167 (1.898, 9.146)***
Income (751–1,200)	3.727 (2.503, 5.518)***	1.425 (0.931, 2.182)	4.413 (2.924, 6.660)***	2.953 (2.139, 4.076)***	2.635 (1.945, 3.570)***	1.364 (1.025, 1.815)*	2.164 (1.557, 3.007)***	2.699 (2.035, 3.578)***	4.025 (2.029, 7.983)***	1.025 (0.726, 1.446)	1.709 (1.237, 2.361)***

OR, Odds Ratio; CI 95%, Confidence Interval for OR. SNA, Social Network Addiction. *p ≤ 0.050, **p ≤ 0.010. ***p ≤ 0.001. Boldface indicates statistically significant odds ratios (*p* ≤ 0.05).

The results indicated significant differences between adolescents with typical development (TD) and those with non-typical development (NTD) in various forms of bullying, cyberbullying, and social network addiction. Adolescents with NTD were more likely to engage in verbal bullying perpetration [*OR* = 2.114, 95% CI (1.397, 3.198), *p* < 0.001], while they showed lower odds of social bullying perpetration [*OR* = 0.495, 95% CI (0.250, 0.980), *p* < 0.05] and physical bullying perpetration [*OR* = 0.350, 95% CI (0.196, 0.624), *p* < 0.001]. However, no statistically significant group difference was observed in total bullying perpetration [*OR* = 1.213, 95% CI (0.809, 1.819), *p* > 0.05]. Similarly, adolescents with NTD had higher odds of experiencing verbal [*OR* = 2.383, 95% CI (1.584, 3.584), *p* < 0.001] and social victimization [*OR* = 2.802, 95% CI (1.891, 4.152), *p* < 0.001], but no significant difference was observed in physical victimization [*OR* = 1.021, 95% CI (0.665, 1.567), *p* > 0.05]. At the aggregate level, adolescents with NTD had significantly higher odds of total bullying victimization than their TD peers [*OR* = 2.341, 95% CI (1.529, 3.584), *p* < 0.001]. Additionally, adolescents with NTD did not show significant differences in the odds of engaging in cyberbullying perpetration compared to their TD peers [*OR* = 0.632, 95% CI (0.300, 1.331), *p* > 0.05]. However, adolescents with NTD had significantly lower odds of being cyberbullied [*OR* = 0.090, 95% CI (0.028, 0.287), *p* < 0.001] and had lower odds of meeting the study criterion for high social network addiction scores [*OR* = 0.548, 95% CI (0.335, 0.897), *p* < 0.05].

Regarding the covariates, age was significantly associated with certain behaviors. Specifically, adolescents aged 15 (compared to those aged 14) showed higher odds of social bullying perpetration and lower odds of physical victimization and being cyberbullied. Gender did not show significant associations with any of the behaviors analyzed.

Furthermore, parental education level and household income were significantly associated with various forms of bullying, cyberbullying, and social network addiction. Overall, adolescents with higher parental education levels were less likely to engage in bullying and cyberbullying behaviors and to meet the study criterion for high social network addiction scores. On the other hand, adolescents from lower-income households exhibited higher odds of engaging in bullying and cyberbullying behaviors, experiencing bullying, and meeting the study criterion for high social network addiction scores.

### Indirect associations between traditional bullying and cyberbullying outcomes through social network addiction

Spearman’s rank-order correlations indicated significant positive associations among all forms of traditional bullying (physical, verbal, relational), cyberbullying (perpetration and victimization), and social-network addiction. Correlation magnitudes ranged from *rho* = 0.14 to *rho* = 0.58 (*df* = 1,456, all *p* < 0.001), indicating small-to-large positive associations across the study variables (see Table 4).

**Table 4:** Descriptive statistics and correlation matrix among study variables (*N* = 1,458).

Variable	*M*	*SD*	1	2	3	4	5	6	7	8	9	10	11
1. Verbal Perpetration (APRI)	8.55	4.01	**–**										
2. Social Perpetration (APRI)	7.39	2.78	0.504***										
3. Physical Perpetration (APRI)	8.01	3.53	0.582***	0.459***	**–**								
4. Total Perpetration (APRI)	23.95	9.09	0.847***	0.716***	0.816***	**–**							
5. Verbal Victimization (APRI)	10.55	5.57	0.348***	0.322***	0.211***	0.357***	**–**						
6. Social Victimization (APRI)	10.35	5.89	0.266***	0.238***	0.135***	0.255***	0.746***	**–**					
7. Physical Victimization (APRI)	8.60	4.56	0.312***	0.268***	0.245***	0.361***	0.492***	0.495***	**–**				
8. Total Victimization (APRI)	29.50	14.60	0.371***	0.322***	0.243***	0.383***	0.904***	0.872***	0.702***	**–**			
9. Cyberbullying (RCBI-II)	11.15	3.01	0.495***	0.403***	0.358***	0.471***	0.355***	0.254***	0.254***	0.360***	**–**		
10. Cybervictimization (RCBI-II)	12.15	3.92	0.330***	0.352***	0.220***	0.375***	0.250***	0.231***	0.265***	0.299***	0.436***	**–**	
11. Social Network Addiction	21.70	12.86	0.429***	0.334***	0.351***	0.451***	0.306***	0.289***	0.238***	0.341***	0.491***	0.378***	**–**

M, Mean; SD, Standard Deviation; APRI, Adolescent Peer Relations Instrument; RCBI-II, Revised Cyber Bullying Inventory-II. ***p ≤ 0.001.

Given the statistically significant bivariate correlations among study variables, a path analysis was estimated to examine the hypothesized direct and indirect associations between traditional bullying, social network addiction, and cyberbullying outcomes. The ordering of the variables represented the hypothesized statistical model; because the data were cross-sectional, it should not be interpreted as evidence of temporal or causal ordering. Age, parental education (mother and father), and family income were included as covariates. The model demonstrated excellent fit to the data [*χ*^2^(10) = 16.662, *p* = 0.082, *CFI* = 0.995, *RMSEA* = 0.030, 90% CI (0.000, 0.055), *SRMR* = 0.009].

The estimated associations were examined separately for adolescents with typical development (TD) and adolescents with non-typical development (NTD). Different descriptive patterns of association were observed among adolescents with TD and those with NTD. Among adolescents with TD, traditional bullying perpetration and victimization were positively associated with social network addiction (perpetration: *β* = 0.363, *p* < 0.001; victimization: *β* = 0.130, *p* < 0.001). Both bullying perpetration and victimization were also positively associated with cyberbullying perpetration (perpetration: *β* = 0.451, *p* < 0.001; victimization: *β* = 0.106, *p* = 0.001) and cybervictimization (perpetration: *β* = 0.188, *p* = 0.001; victimization: *β* = 0.266, *p* = 0.001). Social network addiction was positively associated with both cyberbullying perpetration (*β* = 0.145, *p* < 0.001) and cybervictimization (*β* = 0.163, *p* < 0.001). Additionally, the model-estimated correlation between bullying perpetration and victimization was statistically significant (*r* = 0.323, *p* < 0.001), as was the correlation cyberbullying and cybervictimization (*r* = 0.253, *p* < 0.001).

Bootstrapped estimates indicated statistically significant positive indirect associations through social network addiction among adolescents with TD (see Table 5). Bullying perpetration was indirectly associated with both cyberbullying perpetration [*β* = 0.053, *p* = 0.003, 95% CI (0.017, 0.088)] and cybervictimization [*β* = 0.059, *p* < 0.001, 95% CI (0.031, 0.083)]. Similarly, bullying victimization showed statistically significant indirect associations with cyberbullying perpetration [*β* = 0.019, *p* = 0.011, 95% CI (0.003, 0.034)] and cybervictimization [*β* = 0.021, *p* = 0.003, 95% CI (0.007, 0.036)]. These findings are consistent with partial statistical mediation in the specified cross-sectional model but do not demonstrate temporal or causal mediation.

**Table 5:** Indirect associations between traditional bullying and cyberbullying outcomes through problematic social network use.

Indirect effect	Adolescents with TD			Adolescents with NTD		
	*β*	*p*	*95% CI* (bias-corrected bootstrap)	*β*	*p*	*95% CI* (bias-corrected bootstrap)
Bullying perpetration - > SNA- > Cyberbullying	0.053	0.003	0.017, 0.088	−0.008	0.904	−0.059, 0.053
Bullying perpetration - > SNA- > Cybervictimization	0.059	< 0.001	0.031, 0.083	0.061	0.206	−0.054, 0.175
Bullying victimization - > SNA- > Cyberbullying	0.019	0.011	0.003, 0.034	−0.008	0.904	−0.157, 0.140
Bullying victimization- > SNA- > Cybervictimization	0.021	0.003	0.007, 0.036	0.166	0.232	−0.167, 0.499

SNA, Social Network Addiction.

Among adolescents with NTD, both bullying perpetration (*β* = 0.211, *p* = 0.011) and bullying victimization (*β* = 0.579, *p* < 0.001) were positively associated with social network addiction. Bullying perpetration was positively associated with cyberbullying perpetration (*β* = 0.605, *p* < 0.001), whereas bullying victimization was negatively associated with cybervictimization (*β* = −0.257, *p* < 0.001). No statistically significant associations were found from bullying perpetration and cybervictimization (*β* = 0.110, *p* = 0.311) or between bullying victimization and cyberbullying perpetration (*β* = −0.133, *p* = 0.116).

Furthermore, social network addiction was not significantly associated with either cyberbullying perpetration (*β* = −0.014, *p* = 0.905) or cybervictimization (*β* = 0.287, *p* = 0.200). Accordingly, no statistically significant indirect associations between traditional bullying and cyberbullying outcomes through social network addiction were observed among adolescents with NTD (see Table 5). Finally, the model-estimated correlation between bullying perpetration and bullying victimization was positive and statistically significant (*r* = 0.209, *p* = 0.045), whereas the model-estimated residual correlation between cyberbullying perpetration and cybervictimization was not statistically significant (*r* = 0.059, *p* = 0.304).

## Discussion

### The prevalence of bullying, cyberbullying, and social network addiction

The first research question addressed the prevalence of traditional bullying, cyberbullying, and social network addiction. Overall, 35.9% of participants reported engaging in at least one form of bullying perpetration, while 53.5% reported having been victims of bullying. These rates are considered high in light of international estimates. For example, global data from the Health Behavior in School-aged Children (HBSC) study (Inchley et al., 2020) and UNESCO (2019) reports typically cite traditional bullying perpetration rates ranging from 10 to 30% among adolescents. Thus, the perpetration rate observed in this study exceeds these international benchmarks, suggesting a substantial prevalence of bullying behavior within this population.

The relatively high prevalence of bullying observed in the present study should be interpreted in light of the broader Omani and Arab sociocultural context, while avoiding causal conclusions about factors that were not directly measured. Previous literature has suggested that bullying in Arab and Omani school contexts may be shaped by cultural values, peer dynamics, school environment, and barriers to help-seeking (Kazarian and Ammar, 2013; Kooli, 2019; Al Omari et al., 2022). For example, norms related to authority and seniority, hierarchical relationships within schools, and reluctance to discuss personal or emotional difficulties have been proposed as potentially relevant contextual factors. However, the present study did not operationally assess perceived authority norms, school hierarchy, stigma, help-seeking attitudes, or school climate. Therefore, these factors should be regarded as possible contextual hypotheses rather than evidence-supported explanations of the prevalence rates observed in this sample. Future studies should directly examine whether help-seeking barriers and perceived school climate contribute to the reporting, persistence, or normalization of bullying behaviors.

Furthermore, the victimization rate appears particularly elevated and is consistent with findings from prior research conducted in Oman (Al-Saadoon et al., 2014; Biswas et al., 2020; Fleming and Jacobsen, 2010). This heightened prevalence of victimization is consistent with previous studies conducted in Oman. Although broader sociocultural factors, such as collectivist norms, hierarchical school structures, help-seeking barriers, and limited mental health resources, may be relevant to understanding bullying in this context, these variables were not directly assessed in the present study. Consequently, they should be interpreted as possible directions for future research rather than as explanations supported by the current data.

Despite the unique context of Oman, low levels of peer and parental support (Biswas et al., 2020) and teacher support (Yang et al., 2023) have been consistently associated in the literature with a higher risk of bullying victimization. This pattern suggests that, regardless of cultural differences, supportive relationships play a universally protective role in adolescent well-being. Adolescents who experience strong emotional backing from friends and active involvement from parents are likely to be more resilient when facing bullying and may feel more empowered to report or confront such experiences.

As anticipated, verbal bullying emerged as the most commonly reported form of both perpetration and victimization. This finding is in line with previous studies from Oman (Al-Saadoon et al., 2014) and is also consistent with patterns reported in Western contexts (Inchley et al., 2020).

Regarding cyberbullying, 9.7% of adolescents reported engaging in cyberbullying perpetration, while 19.9% reported being victims. These rates are comparable to recent data from Western countries, where 12% reported cyberbullying perpetration and 16% reported victimization (Cosma et al., 2024) and fit within the wide range reported across the Arab world, where victimization rates vary from 14.8% (Farooq et al., 2023) to 82% (Heiman and Olenik-Shemesh, 2016).

Compared to other Arab populations, Omani adolescents appear to experience a moderate level of exposure to cyberbullying risks. This may be attributed to Oman’s relatively recent entry into the digital age, which, while rapidly advancing, has not yet reached the saturation levels seen in more digitally mature neighboring countries (Al-Badi et al., 2016; Saini and Al-Mamri, 2019). As digital infrastructure, internet accessibility, and social media usage continue to grow, so too may the potential risks associated with online interactions, including cyberbullying.

Finally, nearly one-third of participants (28.5%) exhibited high scores on social network addiction. This finding is consistent with global concerns regarding problematic social media use among adolescents. In the Omani context, the rapid expansion of access to smartphones and social networking platforms (Al-Badi et al., 2016; Saini and Al-Mamri, 2019), combined with limited public discourse around digital wellbeing, may be contributing factors. Given the increasing centrality of social media in adolescents’ lives, particularly in collectivist societies where online interactions reinforce social belonging, this trend warrants attention from educators and policymakers.

### The comparison between adolescents with typical development and those with non-typical development

The second research question explored whether there were differences between adolescents with TD and NTD in terms of perpetration and victimization in bullying and cyberbullying, as well as in social network addiction. Logistic regression analyses revealed significant differences between the two groups in multiple areas.

Adolescents with NTD were more likely to engage in verbal bullying perpetration compared to their TD peers. This finding aligns with previous research from Western literature (Abregú-Crespo et al., 2024), which suggests that young people with neurodevelopmental or psychiatric conditions are at an increased risk of being involved in bullying perpetration. One possible interpretation is that the social challenges experienced by some adolescents within the NTD group may be associated with frustration that is expressed through verbal aggression. While these adolescents may experience difficulties with more complex social interactions,challenges that could limit their involvement in other forms of bullying, such as social or physical bullying,their verbal aggression remains pronounced. Indeed, our results indicate that adolescents with NTD were less likely to engage in social and physical bullying perpetration. This pattern may reflect the greater difficulties that some adolescents within the NTD group experience when navigating complex social interactions, which are often involved in relational and physical bullying. However, this result does not align with the findings in Western literature (Abregú-Crespo et al., 2024). This discrepancy may be attributed to both contextual and practical factors. First, adolescents with neurodevelopmental disorders (NTD) may face social isolation and have fewer opportunities to participate in peer groups where bullying behavior typically occurs (Shattuck et al., 2011). Second, many adolescents with NTD struggle with social communication and interpreting social cues (Baribeau et al., 2015), which can hinder their ability to engage in the complex social interactions necessary for bullying. Third, individuals with NTD are often supported by caring adults and are placed in structured environments that promote positive social interactions and conflict resolution (Berglund Melendez et al., 2020). These environments can help prevent the development of aggressive behaviors. However, these factors were not assessed in the present study and should therefore be regarded as hypotheses for future research rather than explanations supported by the current data.

Consistent with previous research, adolescents with NTD were more likely to be victims of bullying, particularly verbal and social forms of bullying. This aligns with existing literature that suggests adolescents with psychological disabilities or special education needs are at an increased risk for bullying victimization due to their social difficulties, lack of reciprocal friendships, and challenges in peer interactions (Humphrey and Hebron, 2015; Simmons and Antshel, 2021). Research focusing on youth with ID has identified poor social skills, such as social withdrawal and problems, as well as behavior issues, as the strongest predictors of bullying risk (Christensen et al., 2012). Additionally, emotional and interpersonal difficulties are closely linked to victimization in this group (Reiter and Lapidot-Lefler, 2007). For individuals with ASD, communication challenges significantly increase the likelihood of being victimized (Cappadocia et al., 2012). Generally, youth with NTD tend to have fewer reciprocal friendships compared to their typically developing peers (Orsmond et al., 2004). For instance, Solish et al. (2010) reported that adolescents with ID participate less in social activities, have fewer friendships, and experience greater loneliness, often forming fewer complex friendships. These social challenges may contribute to increased rates of bullying victimization and related issues like depression and anxiety (Zeedyk et al., 2014).

These findings should nevertheless be interpreted within the context of the heterogeneous composition of the NTD group. Although all participants included in this group had a formally documented neurodevelopmental condition, the group encompassed adolescents with diverse developmental profiles, including autism spectrum disorder, attention-deficit/hyperactivity disorder, specific learning disability, childhood-onset fluency disorder (stuttering), and other specified neurodevelopmental conditions. These conditions differ substantially in the developmental mechanisms through which they influence social communication, executive functioning, emotional regulation, and peer relationships (American Psychiatric Association, 2022; Maïano et al., 2016; Thapar et al., 2017). Consequently, the observed group differences should be interpreted as aggregate estimates across a diagnostically heterogeneous population. They may reflect vulnerabilities that are shared across neurodevelopmental conditions as well as diagnosis-specific mechanisms that could not be disentangled within the present study.

Interestingly, no significant difference was observed in physical bullying victimization between the two groups. This finding may indicate that adolescents within the NTD group were more likely to experience verbal and social forms of victimization than physical victimization, although the underlying mechanisms remain unclear.

No statistically significant differences were found between adolescents with TD and NTD in cyberbullying perpetration. In contrast, adolescents with NTD showed lower odds of meeting the study criterion for cybervictimization. This finding was unexpected and differs from previous research suggesting that young people with neurodevelopmental conditions may be particularly vulnerable to cybervictimization (Abregú-Crespo et al., 2024; Beckman et al., 2020). It should also be interpreted cautiously because only three adolescents in the NTD group met the criterion for cybervictimization, resulting in a very small number of events on which the estimate was based. Furthermore, the diagnostic heterogeneity of the NTD group should be considered when interpreting this finding, as different neurodevelopmental conditions are characterized by distinct patterns of online social engagement, peer interaction, and digital communication.

Previous studies have documented differences in patterns of Internet and social media use in some neurodevelopmental groups, particularly adolescents with ASD (Kuo et al., 2014; Alhujaili et al., 2022). However, this evidence does not establish that lower online exposure explains the present finding. The study did not assess time spent online, number and quality of online peer relationships, platforms used, parental monitoring, or participants’ ability to recognize and report cyberbullying experiences. Therefore, lower online exposure, differences in the recognition or reporting of cyberbullying experiences, the heterogeneous diagnostic composition of the NTD group, and contextual characteristics of the participating schools should all be regarded as hypotheses requiring empirical examination rather than explanations supported by the present data.

Finally, adolescents with NTD also exhibited lower odds of meeting the study criterion for high social network addiction scores than their TD peers. This finding did not support Hypothesis 4 and should be interpreted cautiously. Previous research has reported comparable amounts of social media use among adolescents with and without ASD, while also identifying possible differences in preferred platforms and motivations for use (Alhujaili et al., 2022; Alon-Tirosh and Meir, 2023). However, these findings were derived primarily from samples of adolescents with ASD and therefore cannot readily be generalized to the diagnostically heterogeneous NTD group examined in the present study. Moreover, differences in amount or purpose of social media use do not necessarily correspond to differences in problematic or addictive use.

The present study did not assess motivations for social media use, types of platforms used, objective time online, parental monitoring, access restrictions, or the educational support received by participants. It is therefore not possible to determine why adolescents with NTD were less likely to meet the study criterion.

Accordingly, the observed group difference may represent genuine variation in problematic social media use, but it may also reflect the heterogeneous composition of the NTD group, contextual characteristics of the participating schools, differences in self-reporting, or the use of a sample-specific percentile threshold. The present data do not allow these alternative interpretations to be distinguished.

Contrary to Hypothesis 5, gender was not significantly associated with any of the bullying, cyberbullying, or problematic social network use outcomes after adjustment for developmental status, age, parental education, and household income. Thus, the expected higher odds of physical bullying perpetration among boys and of relational bullying perpetration and cybervictimization among girls were not observed. This finding differs partly from recent international HBSC evidence showing that boys generally report higher rates of school bullying and cyberbullying perpetration, whereas gender differences in victimization are smaller and vary across countries and forms of involvement (Cosma et al., 2024). Nevertheless, the absence of a significant association between gender and cybervictimization in the present study is consistent with findings from a school-based survey conducted in Dubai, which found no significant association between sex and self-reported electronic bullying victimization (Farooq et al., 2023). The absence of gender differences in elevated problematic social network use should also be interpreted cautiously, given that estimates of problematic use vary substantially according to the classification criteria and cultural context considered (Cheng et al., 2021). These findings suggest that gender patterns may depend on the specific outcome assessed, the cultural context, the measurement and classification procedures employed, and the covariates included in the analyses.

### Indirect associations between traditional bullying and cyberbullying outcomes through social network addiction

The third research question examined whether traditional bullying perpetration and victimization were indirectly associated with cyberbullying outcomes through social network addiction. The findings describe different patterns of direct and indirect associations among adolescents with TD and those with NTD. Because the data were collected at a single time point, these patterns should not be interpreted as evidence of underlying temporal or causal mechanisms. Our findings provide novel evidence regarding the direct and indirect associations linking traditional bullying, problematic social network use, and cyberbullying outcomes, and show that these patterns of association differed between adolescents with TD and those with NTD.

Among adolescents with TD, traditional bullying perpetration and victimization were directly associated with cyberbullying outcomes and also showed statistically significant indirect associations through social network addiction. Because bullying, social network addiction, and cyberbullying were measured concurrently, it cannot be determined whether involvement in traditional bullying preceded problematic social media use or cyberbullying involvement. One possible interpretation, requiring confirmation through longitudinal research, is that adolescents involved in offline bullying may also use social media problematically and may be involved in online aggression or victimization. This interpretation is compatible with displacement and social compensation theories (Kraut et al., 2002), but the present findings do not provide a direct test of the temporal processes proposed by these theories.

Among adolescents with NTD, the indirect associations through social network addiction were not statistically significant. Although bullying perpetration and victimization were positively associated with social network addiction, social network addiction was not significantly associated with either cyberbullying outcome. Regarding the direct associations, bullying perpetration was positively associated with cyberbullying perpetration, whereas bullying victimization was negatively associated with cybervictimization. In contrast, bullying perpetration was not significantly associated with cybervictimization, and bullying victimization was not significantly associated with cyberbullying perpetration. This cross-sectional pattern may be compatible with differences in digital social engagement or online interaction among adolescents with neurodevelopmental conditions; however, the present study did not assess the processes or motivations that might explain these associations. Previous studies have reported that adolescents with NTD, particularly those with autism spectrum disorder or ADHD, may experience difficulties in navigating online social interactions (Alhujaili et al., 2022; Humphrey and Hebron, 2015; Murray et al., 2021). Such characteristics could be relevant to the pattern observed in the present study, although this interpretation remains speculative and was not directly tested.

Moreover, the absence of significant indirect associations through social network addiction in the NTD group may raise the possibility that the psychological functions and motivations underlying social media use differ between adolescents with TD and those with specific neurodevelopmental conditions. However, the present study did not assess motivations, styles of online engagement, or diagnosis-specific patterns of social media use. In addition, the absence of statistically significant indirect associations may partly reflect the relatively small and diagnostically heterogeneous NTD group. Therefore, no conclusions can be drawn regarding whether adolescents with NTD use digital environments in more passive, avoidant, instrumental, or socially oriented ways. These possibilities should be examined in future studies using larger diagnosis-specific samples and more detailed measures of online engagement.

Taken together, these results underscore the importance of tailoring prevention and intervention strategies to the specific needs and behavioral profiles of adolescents with differing developmental statuses. For youth with TD, problematic social media use may be a relevant correlate to consider in interventions that jointly address offline and online aggression. However, the present findings do not demonstrate that reducing problematic social media use would interrupt or reduce cyberbullying involvement. In contrast, for adolescents with NTD, interventions should focus more on strengthening offline protective factors and social skills to mitigate risks of both victimization and maladaptive coping.

### Limitations

The present findings should be interpreted in light of several methodological considerations. First, although the study included a relatively large sample of adolescents, participants were recruited through convenience sampling from only six public schools located in the urban Governorate of Muscat. Consequently, the findings may not generalize to adolescents attending private schools, schools located in rural governorates, or other educational settings in Oman, where educational resources, student composition, socioeconomic characteristics, access to digital technologies, school environments, and peer experiences may differ. Furthermore, although Oman shares important cultural and linguistic characteristics with other Arab countries, differences in educational systems, economic development, and sociocultural norms warrant caution when generalizing the findings beyond the Omani context. Future studies should recruit nationally representative samples including public and private schools across urban and rural settings to strengthen the external validity of the findings.

Second, the cross-sectional nature of the study precludes conclusions regarding temporal ordering and causality. Although the proposed model identified statistically significant indirect associations through social network addiction, these findings should not be interpreted as evidence of causal mediation. Because all variables were assessed at a single time point, alternative developmental pathways, reciprocal influences and the contribution of unmeasured third variables remain plausible. For instance, problematic social media use may increase involvement in cyberbullying, cyberbullying experiences may promote maladaptive online engagement, or both may emerge from broader psychosocial vulnerabilities. Longitudinal studies with repeated assessments and experimental or quasi-experimental designs are required to establish developmental trajectories and clarify causal mechanisms.

Third, the operationalization of the NTD group deserves careful consideration. Although all adolescents included in this group had a formally documented neurodevelopmental condition verified through official school records and Ministry of Education procedures, the NTD group represented a diagnostically heterogeneous population, comprising adolescents with autism spectrum disorder, attention-deficit/hyperactivity disorder, learning disabilities, childhood-onset fluency disorder (stuttering), and other specified neurodevelopmental conditions. While these conditions share atypical neurodevelopment as a common characteristic, they differ substantially in the developmental mechanisms through which they influence cognitive functioning, executive processes, communication, emotional regulation, social adaptation and peer relationships (American Psychiatric Association, 2022; Maïano et al., 2016; Thapar et al., 2017). Combining them into a single analytical group may therefore have obscured diagnosis-specific patterns or associations operating differently across diagnostic categories. Consequently, the findings should be interpreted as aggregate estimates across a heterogeneous developmental population rather than as evidence that the observed patterns apply uniformly across all neurodevelopmental conditions. Furthermore, information regarding diagnostic severity, level of support needs, functional impairment, adaptive functioning, psychiatric comorbidities, co-occurring neurodevelopmental diagnoses, and time since diagnosis was unavailable. These factors are likely to influence both offline and online social experiences and may partly account for individual differences within the NTD group. Although diagnosis-specific analyses would have been scientifically informative, the relatively small number of participants within individual diagnostic categories precluded statistically reliable subgroup analyses. Future research should examine these relationships using larger diagnosis-specific samples while simultaneously considering clinical severity, support needs, adaptive functioning and co-occurring psychiatric conditions.

The findings regarding cybervictimization and social network addiction within the NTD group require particular caution. Only a very small number of participants fulfilled the criterion for cybervictimization, increasing the possibility that the corresponding estimates were influenced by sparse data and the specific diagnostic composition of the sample. Moreover, the study did not examine measurement invariance across adolescents with and without neurodevelopmental conditions. Consequently, it cannot be excluded that differences in the interpretation, recognition or reporting of online experiences contributed to the observed results. In addition, potentially relevant explanatory factors, including patterns of online exposure, motivations for social media use, parental monitoring, digital literacy and access restrictions, were not assessed. These variables may represent important mechanisms underlying the unexpected findings observed in the NTD group and should be incorporated into future investigations.

Another limitation concerns the assessment of social network addiction. Although this construct is widely used in the empirical literature and corresponds to the instrument administered in the present study, it is not currently recognized as a formal clinical diagnosis in the Diagnostic and Statistical Manual of Mental Disorders (5th ed., text rev.; American Psychiatric Association, 2022). Furthermore, participants were classified using a sample-specific percentile threshold rather than clinically validated diagnostic criteria. Accordingly, the present findings should be interpreted as reflecting elevated self-reported symptoms of problematic social media use rather than clinically diagnosed addiction.

A related methodological limitation concerns the dichotomization of the study variables. Although categorical operationalizations were useful for estimating the proportion of participants meeting the study criteria and for conducting logistic regression analyses, dichotomization may have reduced statistical information and introduced classification error. Nevertheless, the original continuous scores were retained in the correlational and path analyses.

The exclusive reliance on adolescent self-report questionnaires also represents a limitation. Self-report measures may be influenced by recall bias, social desirability, and individual differences in the interpretation of questionnaire items, particularly when assessing socially sensitive behaviors such as bullying perpetration, cyberbullying, and problematic social media use. The absence of information from parents, teachers, peers, or school records prevented triangulation of participants’ reports and may have affected the precision of the estimates. Future studies should integrate multiple informants with behavioral and administrative indicators to obtain a more comprehensive understanding of adolescents’ peer experiences.

Finally, several contextual variables that may contribute to bullying and digital behavior were not included in the present study. School climate, teacher and peer support, friendship quality, peer acceptance, and social inclusion were not assessed. Likewise, culturally relevant constructs discussed in the interpretation of the findings, including authority norms, hierarchical school relationships, perceived stigma, and help-seeking attitudes, were not operationalized. Furthermore, digital engagement was measured exclusively through self-report, whereas objective indicators such as screen time, frequency and duration of social media use, platform preferences, and patterns of online interaction were unavailable. The omission of these contextual and behavioral variables leaves open the possibility of residual confounding and limits the interpretation of several observed associations.

Notwithstanding these limitations, the present study provides one of the first comprehensive investigations of bullying, cyberbullying, and social network addiction among adolescents with typical development and the NTD group within the Omani educational context. By examining these interrelated phenomena in an understudied cultural setting and including adolescents with documented neurodevelopmental conditions, the study extends the limited evidence available from the Arab region and offers a foundation for future longitudinal, diagnosis-specific, and cross-cultural research. In particular, the findings concerning the NTD group, especially those relating to cybervictimization and social network addiction, should be regarded as preliminary until replicated in larger, clinically better-characterized, and methodologically richer samples.

## Conclusion, implications, and future directions

The findings of the present study underscore the high prevalence of both traditional bullying and cyberbullying among adolescents in Oman, with critical implications for educational institutions, mental health professionals, and families. The results reveal that both forms of aggression remain pressing concerns within school environments and beyond, necessitating evidence-based preventive and intervention strategies that reflect the complexity of adolescents’ psychosocial development and their interactions within digital ecosystems. In particular, the data emphasize that cyberbullying is not an isolated phenomenon but often overlaps with offline bullying, reinforcing the need for comprehensive responses that address both contexts simultaneously.

For schools and educational policymakers, the outcomes highlight the importance of implementing scientifically validated anti-bullying programs that explicitly target both face-to-face and online forms of victimization. These programs should be grounded in empirical models of adolescent development, incorporate components of social–emotional learning, and be embedded within the formal curriculum to ensure universal access. Such programs are consistent with recent reforms in the Omani education system, which increasingly acknowledge the importance of students’ psychological well-being and emphasize a holistic model of development that integrates cognitive, emotional, and social domains (Al Nasiri et al., 2022). In practice, this means that school staff must receive formal training in recognizing subtle and non-verbal signs of bullying, particularly when dealing with adolescents who may have communication challenges, such as those with neurodevelopmental traits. Further, administrators should establish transparent protocols for monitoring, reporting, and responding to bullying incidents in both physical and digital contexts.

From a clinical perspective, the results suggest that assessments of adolescent mental health should include standardized screening tools for bullying and cyberbullying exposure. Clinical psychologists and school counselors must be equipped to identify the psychological sequelae of both victimization and perpetration, which may include symptoms of anxiety, depression, aggression, and withdrawal. Importantly, among adolescents with TD, the study identified statistically significant cross-sectional indirect associations between traditional bullying and cyberbullying outcomes through social network addiction. These findings indicate that offline bullying involvement, problematic social media use, and cyberbullying involvement are interrelated; however, they do not demonstrate that problematic social media use temporally or causally contributes to a transition from offline to online aggression. Accordingly, problematic social media use may be considered as one relevant correlate within broader assessment and prevention programs. Such programs could incorporate components focused on digital self-regulation, emotional competence, and peer relationship management; however, these intervention components should be empirically evaluated and tailored to the specific cognitive and emotional needs of adolescents. Longitudinal research is also needed before drawing conclusions about the role of problematic social media use as a mechanism of change.

For adolescents with NTD, the findings suggest that traditional interventions may be insufficient. Their higher likelihood of verbal and social offline victimization supports the implementation of specialized programs offering structured opportunities to develop interpersonal skills in realistic, peer-based settings. Although the NTD group reported lower cybervictimization in the present sample, this unexpected finding requires replication and should not be interpreted as demonstrating reduced vulnerability to online harm. Programs should include training in specific social interaction strategies, such as initiating conversations, giving and receiving help, interpreting social cues, and managing conflicts. These activities must be supervised by trained facilitators and include repeated opportunities for guided practice, role-play, and immediate feedback. Interventions should also incorporate structured opportunities for adolescents with NTD to interact with neurotypical peers in a supportive and inclusive environment. Such peer-mediated approaches have demonstrated efficacy in improving social competence and reducing stigma in similar populations. Furthermore, educators should receive specific guidance in identifying the subtle, and often overlooked, indicators of bullying in students with communication or cognitive differences.

The study also points to the need for increased family engagement in addressing bullying and cyberbullying. Parents and guardians should be informed about the higher likelihood of verbal and social offline victimization observed among adolescents with NTD in the present study. At the same time, the unexpected lower rate of self-reported cybervictimization should be interpreted cautiously and should not lead families or schools to reduce attention to online safety. Families should be encouraged to maintain open communication with their children, regularly monitor digital activity, and seek out school-based or community-based support services when concerns arise (Good and Fang, 2015). Parental advocacy within educational institutions is essential to ensure that adequate individual supports, such as counseling or classroom accommodations, are provided in a timely and culturally sensitive manner. Importantly, families of adolescents with and without NTD should be involved in joint school-based programs that promote awareness, empathy, and collective responsibility for a safe school environment.

In conclusion, the present study provides a nuanced understanding of the interplay between traditional bullying, cyberbullying, and social network addiction among adolescents in Oman. It contributes to a growing body of literature showing that adolescents’ experiences of aggression are associated with multiple factors, including digital behaviors and neurodevelopmental characteristics. The results emphasize the urgent need for comprehensive, multi-tiered interventions that involve schools, families, and mental health professionals. Future research should employ longitudinal designs to clarify temporal ordering and examine the potential processes underlying these associations. Experimental or quasi-experimental research would be needed to evaluate causal mechanisms and the efficacy of targeted interventions (Al Asmi and Caldwell, 2018). Particular attention should be paid to how cultural values, social norms, and digital infrastructures influence both the expression and mitigation of bullying. Further studies are also needed to develop and test intervention models specifically adapted to adolescents with neurodevelopmental differences, in order to improve their resilience, enhance their social integration, and reduce their risk of victimization in both offline and online settings.

## Data Availability

The original contributions presented in the study are included in the article/supplementary material, further inquiries can be directed to the corresponding authors.
